# Application of Non-Thermal Plasma to Fungal Resources

**DOI:** 10.3390/jof8020102

**Published:** 2022-01-21

**Authors:** Mayura Veerana, Nannan Yu, Wirinthip Ketya, Gyungsoon Park

**Affiliations:** 1 Plasma Bioscience Research Center, Department of Plasma-Bio Display, Kwangwoon University, Seoul 01897, Korea; mayuraveerana@gmail.com (M.V.); nannan19950326@163.com (N.Y.); pam7794p@gmail.com (W.K.); 2 Department of Electrical and Biological Physics, Kwangwoon University, Seoul 01897, Korea

**Keywords:** non-thermal plasma, fungi, inactivation, activation, reactive species

## Abstract

In addition to being key pathogens in plants, animals, and humans, fungi are also valuable resources in agriculture, food, medicine, industry, and the environment. The elimination of pathogenic fungi and the functional enhancement of beneficial fungi have been the major topics investigated by researchers. Non-thermal plasma (NTP) is a potential tool to inactivate pathogenic and food-spoiling fungi and functionally enhance beneficial fungi. In this review, we summarize and discuss research performed over the last decade on the use of NTP to treat both harmful and beneficial yeast- and filamentous-type fungi. NTP can efficiently inactivate fungal spores and eliminate fungal contaminants from seeds, fresh agricultural produce, food, and human skin. Studies have also demonstrated that NTP can improve the production of valuable enzymes and metabolites in fungi. Further studies are still needed to establish NTP as a method that can be used as an alternative to the conventional methods of fungal inactivation and activation.

## 1. Introduction

Fungi are the second-most abundant group of organisms after insects [1] and they play a significant role in agriculture, biomedicine, global health, and industry [2]. The number of fungal species on earth is estimated to be 11.7–13.2 million [3]. Over the last 100 years, the number of pathogenic fungi infecting plants, animals, and humans has increased [4]. Fungal pathogens cause some of the most lethal infectious diseases in humans and animals and fungal infections are responsible for the death of approximately 1.6 million people annually [5]. In the United States, fungal diseases were reported to cause economic losses of more than 7.2 billion dollars in 2017 [6]. The worldwide increase in invasive fungal infections, along with the spread of resistant fungal pathogens, is a serious threat to human health [6]. Fungi also produce toxins that are carcinogenic or are responsible for the decay or contamination of food products. On a global scale, fungal infections are jeopardizing food security by reducing crop yields or by resulting in the death of plants [7].

Despite the fact that some fungi are harmful, many fungi are used in industries, including the food and feed, pharmaceutical, paper and pulp, textile, detergent, and biofuel industries [2]. For centuries, humans have used fungi to ferment their foods. Yeasts are a central part of traditional and modern food manufacturing processes, wherein they are used to degrade waste and synthesize industrially useful products [8]. The benefits of fungal resources can be attributed to the enzymes produced by fungi. Fungal enzymes are associated with advantages, such as catalysis, rapid production and high yield, ease of genetic manipulation, and biodegradability [9]. Enzymes of fungal origin account for almost half of all commercial enzymes [9]. The market for filamentous fungi that produce plant-biomass-degrading enzymes is worth €4.7 billion and is predicted to double in the next ten years [10]. Biological enzymes (or biocatalysts), particularly those derived from microorganisms, have become essential for the rapidly growing biotechnology industry.

As fungi exert both beneficial and harmful effects, it would help to functionally enhance beneficial fungi, while inactivating harmful fungi. Several technologies have been developed and tested to control pathogenic and spoiling fungi and improve the functional aspects of beneficial fungi [11,12]. During the last decade, non-thermal plasma (NTP) has shown great potential as a tool for inactivating pathogenic fungi [13,14,15,16,17]. Recent studies have also shown that NTP can improve the production of valuable fungal constituents, such as enzymes, by beneficial fungi [18,19,20]. In this review, we compile and describe use of NTP to control the growth of harmful fungi, while functionally enhancing beneficial fungi by focusing on research published since 2010.

## 2. NTP Technology

NTP is an ionized gas—the fourth state of matter—that generates reactive chemical species, such as reactive oxygen and nitrogen species, electrons, atoms, neutral molecules, charged species, and ultraviolet radiation [21]. NTP can be artificially generated from ambient air or certain gases at atmospheric and low pressures in the presence of a high voltage (~kV) electric current. Radio frequency (RF) power, Microwave (MW) power, alternating current (AC), or direct current (DC) can be used for plasma discharge [22]. Several configurations of plasma devices have been developed: dielectric barrier discharge (DBD) plasma, plasma jet, corona discharge plasma, and gliding arc discharge plasma [22]. Dielectric barrier discharge (DBD) plasma can be generated between two electrodes separated by a dielectric barrier after a high-voltage DC or AC current at high frequency (~kHz) is applied. In a plasma jet, when working gas passes through two cylinder-type electrodes in which one electrode is connected to an electric power source at high frequency, it is ionized and exits through a nozzle with a jet-like appearance. Corona discharge plasma is generated between two or more needle-type or wire-type electrodes after high voltage is applied. Gliding arc discharge plasma is produced under high voltages at the spot where two electrodes are within a few millimeters of each other.

**Figure 1:**
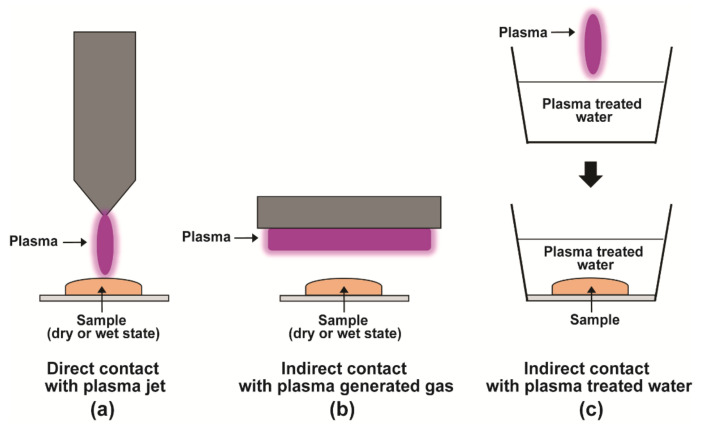
Various strategies used for plasma treatment. Samples can be treated in a dry or wet state. (**a**) The sample is directly exposed to a plasma jet. (**b**) The sample is indirectly exposed to a gas produced from plasma. (**c**) The sample is submerged in plasma-treated water.

The non-thermal nature of NTP has enabled its biological applications [22,23]. NTP has been used in medicine (cancer therapy and wound healing), food industry (microbial decontamination of food), and agriculture (plant disinfection, enhancement of seed germination, and plant growth). The impact of the plasma treatment can be varied by modulating the voltage, treatment time, and gases used for plasma generation [22]. Various strategies are used to subject samples to plasma treatment. These include direct contact with a plasma jet, indirect contact with plasma-generated gas, and indirect contact with plasma-treated water (Figure 1).

The type of sample and the purpose of treatment determine the plasma device and treatment settings used. NTP has demonstrated dual effects (activation and inactivation) on cells, tissues, and organisms depending on the applied dose and species [22]. Plasma generates various levels of reactive oxygen species (ROS) and reactive nitrogen species (RNS) that activate or inactivate cells and tissues (as determined by the dose used) [24]. In medicine, NTP has been used to inactivate microorganisms and cancer cells and to activate cell proliferation and wound healing [25,26]. In agriculture, NTP has been used for disinfecting seeds and fresh produce as well as for enhancing seed germination and plant growth [22]. The dual effects of plasma indicate that it can serve as a promising tool for solving problems in medicine and agriculture.

## 3. Inactivation of Fungi Using NTP

### 3.1. Inactivation of Fungal Spores, Cells, and Biofilms In Vitro

The antimicrobial activities of non-thermal atmospheric and low-pressure plasma have been demonstrated in many studies. The fungicidal effects of NTP can be evidenced by the killing or inactivation of fungal spores and cells in vitro (Table 1).

Although several studies demonstrate that fungi are less sensitive to NTP than bacteria [35], this issue is still controversial and requires more corroboration. NTP features different efficacies with respect to fungal inactivation, and this is dependent on the fungal species targeted, feeding gases, distance between the plasma device and sample, and treatment time. Among yeast-type fungi, *Candida albicans* (human fungal pathogen) and *Saccharomyces cerevisiae* (model yeast) have been targeted using various plasma sources (Table 1). Most studies have reported that NTP can efficiently inactivate yeast cells. Importantly, plasma generated in a sealed package has been reported to effectively inactivate *C. albicans* [43]. Several studies performed using *S. cerevisiae* provide the detailed information on NTP effects on fungal cells [66,67,68]. NTP-generated reactive oxygen species (ROS) caused the accumulation of intracellular ROS and calcium ions (Ca^2+^) and ultimately led to cell apoptosis associated with cell cycle arrest at G1 phase through depolarization of mitochondrial membrane potential and fragmentation of nuclear DNA [68]. The apoptosis of *S. cerevisiae* cells was also observed in the treatment of yeast-contaminated water with NTP [66]. In this study, we found that singlet oxygen (^1^O_2_) among ROS generated in NTP-treated water contributed the most to yeast inactivation [66]. When NTP was applied to yeast cells on an agarose tissue model, the concentration of hydroxyl radical (·OH) and pH were critical for the inactivation efficiency, and the inactivation pattern of yeast cells followed the distribution of ·OH [67]. Studies have also shown that NTP can efficiently inactivate the spores of filamentous fungi, such as *Aspergillus* sp., *Penicillium* sp., *Alternaria* sp., *Byssochlamys nivea*, *Cladosporium sphaerospermum*, *Cordyceps bassiana*, and *Neurospora crassa*, which infect plants and spoil food (Table 1). Further, NTP has been shown to suppress ergosterol biosynthesis and increase keratinase activity in fungi [70]. The antifungal activity of NTP can be synergistically enhanced by using other compounds as shown in the study performed by Fukuda et al. [36]. This research group found that ferrous chloride and ferrous sulfate—from the Fenton reaction—improved the fungicidal effect of plasma against a melanized fungus., *Aureobasidium pullulans* [36].

NTP treatment has been reported to be associated with safety issues in some cases. Microbial strains that survive the action of the plasma are genetically and phenotypically modified, and these modified strains can be environmentally hazardous. Tyczkowska-Sieroń et al. demonstrated that the *C. albicans* that survived after plasma treatment exhibited genetic variation, while not showing any significant changes in metabolism and drug susceptibility [37]. This indicates that NTP treatment is associated with a lower likelihood of generating genetically and phenotypically unfavorable strains [37]. However, more experimental data should be obtained regarding this safety issue. Ma et al. also investigated the safety issue of plasma: the protection of the nearby cells and tissues from plasma-induced oxidative stress [69]. This research group suggested that the elevation of antioxidant gene expression through genetic engineering, the creation of hypoxia condition, or the use of anticancer drugs could be more effective than the extracellular scavenging of reactive species to protect cells and tissues from plasma oxidative damage [69].

Biofilm development is a crucial virulence component for pathogenic fungi because biofilms are protected by a polymeric extracellular matrix (ECM) and are resistant to antifungal agents. NTP has been reported to successfully control the growth of *C. albicans* biofilms (Table 1). In different studies, *C. albicans* biofilm formation was inhibited by certain plasma sources, such as plasma jet or dielectric barrier discharges (DBD), using different gases (helium, argon, oxygen, or mixture of gases) (Table 1). Plasma treatment showed an efficiency that was more than two times better at inhibiting the colonization and formation of *C. albicans* biofilms (compared to chemical treatment methods) [74,77]. Sequential treatment with plasma and antifungal chemicals can eliminate *C. albicans* biofilms more effectively than individual treatments, thereby indicating a synergistic effect [76]. Recently, the prevention of the formation of *Aspergillus flavus* biofilms by direct (gas plasma treatment) and indirect (plasma-activated water, PAW) treatments was reported [71]. In this study, the metabolic activity and spore viability of *A. flavus* were significantly decreased, yielding a maximum reduction of 2.2 log_10_ CFU/mL—with gas plasma treatment—and 0.6 log_10_ CFU/mL (with PAW treatment) [71].

The overall effects of NTP treatments on fungal biofilms are similar to those on bacterial biofilms. They include a significant decrease in cell viability, release of DNA and proteins, membrane lipid peroxidation, and breakdown of cell walls, resulting in impaired cell wall integrity and cell leakage [71,149]. Various reactive oxygen and nitrogen species—short-lived species such as hydroxyl radical (·OH), atomic oxygen (O), superoxide (·O_2_^−^), and singlet oxygen (^1^O_2_), and long-lived species such as hydrogen peroxide (H_2_O_2_), gaseous ozone (O_3_), nitric oxide (·NO), nitrogen dioxide radical (·NO_2_), nitrite (NO_2_^−^), and nitrate (NO_3_^−^)—generated from NTP are responsible for the antifungal effects [72]. In bacteria, hydroxyl radicals, gaseous ozone, and nitric oxide, in particular, are thought to be effective at inactivating biofilms [150,151]. These species may be able to play major roles in fungal biofilm eradication.

### 3.2. Inactivation of Fungi in Agriculture and Foods

Fungi often damage crop plants and spoil foods. NTP is known to inactivate fungal spores and cells in vitro (Table 1). NTP efficiently inactivates fungi associated with crops and food products, and various levels of decontamination and deactivation have been observed (Table 1).

Seeds contaminated with fungi are often subjected to NTP treatment (Table 1), resulting in the eradication of many seed-borne fungal diseases and mycotoxin contamination. Fungicide treatment is the standard method to disinfect contaminated seeds. The emergence of fungicide resistance and concerns about environmental safety have led to the assessment of NTP as an alternative tool to treat seeds. Studies have shown that NTP disinfects seeds contaminated (naturally or artificially) with fungi, and the efficiency of seed disinfection varies among seeds and fungal species (Table 1). Mravlje et al. analyzed the fungal community on RF plasma-treated buckwheat seeds and found a significant reduction in the frequency and diversity of fungal strains [152]. They also found that *Alternaria* and *Epicoccum* species were the most resistant to plasma [152]. NTP also disinfected seeds artificially inoculated with spores of phytopathogenic fungi, such as *Alternaria alternata*, *Aspergillus flavus*, *Aspergillus niger*, *Aspergillus parasiticus*, *Cladosporium* *fulvum*, *Fusarium* *circinatum*, *Fusarium culmorum*, *Fusarium fujikuroi*, *Fusarium oxysporum*, *Penicillium decumbens*, *Penicillium verrucosum*, and *Rhizoctonia solani* [46,78,82,83,85,94,95,96,97,98,99,100]. Although the sensitivity to the plasma was not significantly different among the fungal species, subtle differences were observed. Most of the studies involved the treatment of dry seeds with plasma and several showed differences between dry and wet seed treatments [95,96,97]. Rice seeds contaminated with *F. fujikuroi*, a pathogenic fungus that is responsible for causing rice bakanae disease, were treated with different plasma systems, such as air plasma jet, air DBD plasma, and underwater arc discharge plasma [95,96,97], and although the voltage of the plasma devices was different, these treatments resulted in the *F. fujikuroi*-contaminated rice seeds being disinfected with an efficiency of over 80%, regardless of seed wetness [95,96,97].

Vegetables and fruits are also often targets of plasma disinfection. Fungi speed up the spoilage of products and produce mycotoxins harmful to humans and animals. Controlling fungal contamination is critical for improving the shelf-life and storage of post-harvest fresh produce, as well as food safety. Both artificially and naturally contaminated fruits and vegetables were examined after plasma decontamination [15,101,102,103,104,105,106,107,108,109,110,111,112,113,114,115,116,117,118,119,120]. *Aspergillus* and *Penicillium* species are frequently used for the artificial contamination of fruits and vegetables, and plasma is used before or after fungal inoculation. Plasma treatment eliminated fungal contamination from artificially inoculated fruits and vegetables by 50–100%, depending on the plasma device, air pressure, feeding gases, treatment time, and voltage, regardless of pre-treatment or post-treatment (Table 1). Fungal regrowth was not observed after plasma treatment in many studies for at least a year. Even when fungal regrowth was observed, the level of regrowth was less than that observed in the non-treated control [15]. The wash water of fruits is often contaminated with fungal spores that may pollute the environment. Plasma treatment may help solve this problem. Ouf et al. showed that plasma decreased the number of fungal spores (74.7–100%) in the wash water of cherries inoculated with *Aspergillus niger* and *Penicillium italicum* [107].

DBD plasma or plasma jet treatments effectively removed naturally occurring harmful fungi on blueberries, kumquats, bananas, and grapes [109,111,113,115,116]. In these studies, 25–100% fungal removal was achieved from the surface of fresh produce depending on plasma devices, electric power, treatment time, and feeding gases. Liu et al. developed a plasma equipped refrigerator. They found that bananas and grapes in the refrigerator were preserved for much longer with no elevation of fungal growth on the surface than those stored conventionally [116]. Mung bean sprouts and button mushrooms were decontaminated using plasma-treated water by 0.5 and 2.84 log CFU reduction of fungi, respectively [112,114]. Many studies have shown that the properties of fruits and vegetables were not significantly altered by plasma treatment. However, Lacombe et al. observed a significant reduction in firmness and anthocyanins in blueberries after plasma treatment [115].

Pre-harvest plants are less frequently studied using plasma-mediated fungal disinfection than post-harvest fresh produce. When a plasma jet was directly applied to symptomatic leaves of *Philodendron erubescens* infected with fungi, no further symptom development occurred, and the leaves recovered from the infected state [122]. Inflorescences of medical cannabis inoculated with *Botrytis cinerea* were efficiently disinfected with plasma (5-log reduction in fungal spore CFU number) [121].

NTP has also been applied to the fungal decontamination of processed and packaged foods, and it effectively removed fungal spores. Spores of *A. flavus* on packaged beef jerky were inactivated with an efficiency of 2–3 log CFU/g reduction after plasma treatment [124]. A sealed package of fungal contaminated pistachios was completely decontaminated when the package was placed between laser electrodes for 18 min [125]. The fungal contamination of onion and red pepper powder, brown-rice cereal bars, saffron, and shredded salted kimchi cabbage was reduced by plasma or plasma-treated water. In these studies, fungal spores were successfully removed with an efficiency of 1.5–2.5 log CFU reduction or completely inactivated, and the shelf-life was extended up to 20 days [123,126,127,128,131]. Natural yeast contamination in freshly ground tomato juice was removed by glide-arc type plasma with a maximum 3 log CFU reduction [129].

Mycotoxin-producing fungi present on agricultural products and foods are a threat to human and animal health. Complete or over 90% degradation of mycotoxins, such as AAL (*Alternaria alternata f*. sp. *Lycopersici*) toxin, aflatoxin, deoxynivalenol, enniatins, fumonisin, sterigmatocystin, T2 toxin, trichothecenes, and zearalenone was observed after treatment with NTP for several minutes; the degradation rate varied depending on the chemical structure of the mycotoxin [137,153]. Mycotoxin removal from contaminated agricultural products and foods has been focused on aflatoxins (Table 1). Nuts and cereals contaminated with aflatoxin or aflatoxin-producing fungi were treated with plasma, with aflatoxin B1 being the most frequently targeted. A 50–90% reduction in aflatoxin B1 was observed in plasma-treated nuts and cereals, depending on the type of nut and cereal, plasma source, and treatment time [81,132,133,134,135,136]. Siciliano et al. found that aflatoxin B1 was more sensitive to NTP than aflatoxin B2, G1, and G2 under various plasma treatment conditions [132]. Sen et al. compared the effects of atmospheric and low-pressure plasmas with that of gamma irradiation and found that gamma irradiation was more efficient at eradicating aflatoxin B1 itself and plasma treatment was more efficient at removing aflatoxin B1 from contaminated spiked hazelnuts [133].

Many studies have suggested that fungal decontamination by NTP could result from individual or synergistic actions of reactive oxygen species (ROS) and reactive nitrogen species (RNS) produced by the plasma. ROS and RNS from plasma may erode fungal cells through etching [154], and they react with chemical components of the fungal cell surface, leading to the degeneration of cell walls and membranes [155]. Many studies have also suggested that plasma-generated ROS and RNS could make the physicochemical properties of the surfaces of agricultural products unfavorable for fungi, or even directly inactivate fungal spores [156,157].

### 3.3. Inactivation of Fungi in Medicine

Fungi cause many health problems in humans, notably skin and mucosal infections and allergies. There are approximately 300 pathogenic fungi, also known as medical fungi [158]. Most fungal infections occur in immunocompromised patients in hospitals [159]. Fungal inactivation is essential to prevent cross-infection and the further deterioration of patient health. NTP can kill bacteria and fungi in the air and decompose harmful gases and tiny particles. Therefore, it is used regularly for air disinfection in hospitals [160]. NTP is also used for the disinfection and sterilization of temperature-sensitive medical instruments and fungi-infected tissues [161,162]. NTP is frequently used in fungal skin infections. The commonly targeted fungi for NTP treatment are *Trychophyton* sp., *Candida albicans*, and *Microsporum* sp. (Table 1). *Arthroderma benhamiae* and *Epidermophyton floccosum* are occasionally used in experiments (Table 1). Many studies have demonstrated that *Trichophyton* sp., the fungal species that causes onychomycosis, was eradicated in liquid suspension or on agar media after treatment with certain NTP sources [138,139,141,146,148]. The maximum reduction of *Trychophyton rubrum* in nails was 6 log when treated with a floating electrode DBD plasma [140]. In this study, authors found that the rate of decontamination of *T. rubrum* was faster compared to that of bacteria *Escherichia coli* using the same plasma device [140]. However, plasma jet and surface micro-discharge plasma were more efficient at removing *E. coli* than *T. rubrum* from infected nails [140]. Ali et al. showed that growth of *T. rubrum* and *Trychophyton mentagrophytes* was significantly inhibited in an infected skin model after treatment with a floating electrode DBD plasma [148].

Candidiasis is an infection caused by yeast-type fungi (*Candida* sp.) and usually affects the mouth, genitals, skin, and internal organs. *Candida* cells are highly susceptible to NTP, as demonstrated by in vitro treatment studies [138,142,145,146,147]. Depending on electric plasma pulses, nails infected with *C. albicans* showed a 10× and 100× reduction in fungal viability [138]. Borges et al. observed that *C. albicans* biofilms were significantly eradicated after plasma jet treatment, but not in the infected tongue [145]. However, a histological analysis showed that *Candida* tissue invasion was markedly reduced in plasma-treated samples [145].

A higher level of fungal decontamination occurs with the combination of NTP with other treatment methods. Lux et al. reported that nail plate abrasion, refreshment, and NTP improved fungal removal by 85.7% [139]. The combined treatment with silver nanoparticles and NTP jet decreased the minimum inhibitory concentration of silver nanoparticles [143]. NTP can be combined with other drugs to kill live fungi of some skin diseases, such as body moss and chronic wounds [163].

## 4. Activation of Beneficial Fungi by NTP

NTP is also a new technology for exerting activation effects on many organisms, such as enhancing seed germination and seedling growth, increasing antioxidant enzyme activity, elevating soluble protein and demethylation levels, accelerating wound healing processes, and activating stem cell differentiation [26,164,165]. Compared to the inactivation effect of NTP, the activation of cellular processes in microorganisms, including fungi, has rarely been studied. Many microorganisms are beneficial to humans and used in food, agriculture, medicine, industry, and bioremediation [166,167]. Fungi have also demonstrated their usefulness to humans [2]. Studies have shown that NTP enhanced the functional aspects of beneficial fungi through non-mutational or mutational changes (Table 2).

### 4.1. Activation through Non-Mutational Ways

Approximately 82% of commercial enzymes in food industries are fungal in origin [204], and improving enzyme production in fungi is considered to be essential for many sectors. The efficiency of intracellular expression and the extracellular secretion of enzymes often becomes a technical bottleneck for the large-scale production of fungal enzymes. Several studies have shown that NTP can improve enzyme production in fungi (Table 2). An NTP jet using helium was used to increase the production of recombinant phytase in yeast (*Pichia pastoris*) [18]. Plasma treatment increased the production of recombinant phytase compared to that of the control in a time-dependent manner. In addition, the plasma significantly increased phytase activity by approximately 125% after 4 h. Presumably, the ROS from the plasma modified the protein structure and increased enzyme activity [18].

Our research group showed that spore germination and α-amylase secretion in *Aspergillus oryzae* was enhanced after treatment with a micro-dielectric barrier discharge (micro-DBD) nitrogen plasma, and plasma jet [20,168,169]. We also found that long-lived species (NO_2_^−^ and NO_3_^−^) produced in the media by plasma played a critical role in activating enzyme secretion from fungal hyphae.

### 4.2. Activation through Mutagenesis

Studies have demonstrated that NTP can induce mutations in fungal genomes, improving fungal vitality and functions (Table 2). The “atmospheric and room temperature plasma” (ARTP) mutation system has been actively used for inducing fungal mutations [205]. In the ARTP mutation system, a radio-frequency atmospheric-pressure glow discharge (RF APGD) plasma jet is used, and this plasma produces a high concentration of active, neutral, and charged species under atmospheric pressure using radio frequency power. These species can damage the DNA strands in fungal cells, causing mutations (missense, deletion, or frame shift) through an incomplete process of gene repair [205]. Fungal spores treated with ARTP are cultured, and viable colonies are selected and cultured for generations. Colonies showing improved functions or phenotypes are selected continuously for generations as mutants. Although mutations induced by NTP may be non-usable and risky by-products in some applications, they may be helpful in the strain improvement of beneficial fungi [206].

Several studies (Table 2) have demonstrated that plasma mutagenesis has improved enzyme activities in fungi. Mutant strains of *Trichoderma viride* and *T. reesei* generated by ARTP exhibited an increase in cellulase activity of approximately a twofold [198,199,200]. The B-2 mutant strain of *A. oryzae* showed increased acid protease, neutral protease, and total protease activities at levels of 54.7, 17.3, and 8.5%, respectively [174]. The mutant H8 of *A. oryzae* showed a significant increase in the activities of neutral proteases, alkaline proteases, and aspartyl aminopeptidase during fermentation [19]. ARTP-induced mutants of *A. niger* and *S. cerevisiae* showed improved production of glucoamylase (70% increase) and glutathione synthetases activity (41–72% increase), respectively [172,195]. Similarly, mutants of *P. oxalicum* generated by combined ARTP/EMS mutagenesis revealed a higher production of raw starch-degrading enzymes (61.1% increase) [187].

Mutant strains of yeast-type fungi generated using ARTP demonstrated improved biodiesel and sugar-alcohol production (Table 2). An *R. toruloides* (oleaginous yeast) mutant generated by ARTP showed enhanced tolerance to inhibitors in lignocellulosic hydrolysate. It grew in lignocellulosic hydrolysate and transformed carbohydrates into long-chain fatty acids, thus contributing to biodiesel production [189,190]. This mutant strain elevated the expression level of genes involved in regulating tolerance to stress from lignocellulosic hydrolysate [191]. Several studies showed that the production of sugar alcohols, which are useful in the food, chemical, and pharmaceutical industries, could be increased in ARTP-induced fungal mutants. For example, the ARTP-induced *P. anomala* mutant produced 32.3% more sugar-alcohol than the parent strain [188]. The M53 mutant of *Y. lipolytica* showed an increase in erythritol production from 145.2 g/L to 200 g/L [203]. The mutagenesis of *Candida tropicalis* by ARTP increased xylitol yield by 22% and enhanced xylose reductase’s activity and relative gene expression [181]. The mutant A6 of *C. parapsilosis* showed an increase in the yield of D-arabitol (32.92 g/L) by 53.98% compared to the parent strain [180].

ARTP mutagenesis (Table 2) improved the production of fungal carotenoid, an important bioactive compound used as an anticancer agent, antioxidant, and immune-response stimulant. The production of the carotenoid lycopene was 55% higher in the *Blakeslea trispora* mutant A5 than in the parent strain [177]. The combined use of chemical and ARTP mutagenesis showed increased levels of carotenoids and lipids in the *R. toruloides* XR-2 mutant strain [192]. The K4 mutant strain of *R. mucilaginosa* generated from the same method produced a 121% higher concentration of carotenoids than the original strain [193].

An improved production of organic and fatty acids was found in ARTP-induced fungal mutants. For example, the 1-C6 mutant strain of *Y. lipolytica* produced a significantly higher amount of α-ketoglutaric acid than the wild type (51.8% higher in 500 mL shake flasks and 45.4% higher in a 3 L fermenter) [201]. A combined mutagenesis with ARTP and diethyl sulfate of *Mortierella alpine* produced the D20 mutant that exhibited 40.61% increased yields of arachidonic acid (ARA), and increased the yield of total fatty acids by about 7% [186]. Polymalic acid (PMA) produced from the ARTP-induced *Auerobasidium pullulans* AH-21 mutant was 13.8% higher than that produced by the wild type [176]. The ARTP-induced *A. terreus* mutant AT-90 produced the highest level of itaconic acid [175]. In an *A. oryzae* mutant strain generated by a combined mutagenesis of microwave, UV irradiation, heat-LiCl, and ARTP kojic acid was quantified as approximately 47–292.3% higher than the original strain, and the transcription of the genes related to kojic acid biosynthesis was also enhanced [173]. A transcriptome analysis of *Fusidium coccineum* and its ARTP-mutagenized strains showed that the transcription levels of most genes involved in fusidic acid biosynthesis significantly increased in the mutant strain, leading to the enhanced production of fusidic acid [182]. Luo et al. discovered an ARTP-induced mutant of *C. glabrata* that showed a 32.2% increase in pyruvate levels [178]. Improved polysaccharide production was also reported in medicinal fungi. The polysaccharide content of *Ganoderma lingzhi* was increased 25.6% by ARTP-induced mutants [183]. The yield of fruiting body and polysaccharide in an ARTP-induced mutant of *H. erinaceus* increased by 22% and 16%, respectively [185]. Similarly, in mutants of *S. sanghuang*, polysaccharide yields were significantly increased by 1.2 to 1.5 fold [196].

The improved production of organic compounds in fungi by ARTP has been reported. For example, gluconate production in *A. niger* mutant strains was enhanced by 12.1–32.8% [171]. The yield of pneumocandin B0, a starting molecule for the semi-synthesis of the antifungal drug, echinocandin, was elevated in *G. lozoyensis* mutants by 1.39 to 1.65 fold [184]. Echinocandin B production in *A. nidulans* was also improved by ARTP mutagenesis with a 1.3 fold increase [170]. ARTP mutants of *S. bombicola* enhanced the production of sophorolipids (SLs) used in several applications, such as food, cosmetics, detergent, environmental, petroleum nanotechnology, and pharmaceutical industries [197]. Specific and total SL production in *S. bombicola* mutant strains exhibited an increase of over 30% in lactonic SLs, acidic SLs, and total SL production compared with the wild strain [197]. ARTP mutagenesis was useful in reducing the production of highly toxic methanol by *S. cerevisiae* in brewed wine [194]. The *S. cerevisiae* S12 mutant decreased methanol production by 72.54% [194].

NTP is a useful tool for increasing the production of enzymes and many useful metabolites and compounds in beneficial fungi. However, several factors, such as the type of plasma and fungi, the dosage of plasma, and the RONS (reactive oxygen and nitrogen species) released, are important considerations when evaluating the effects of plasma on the activation of fungi. The majority of current studies focus on using NTP as a mutagenesis tool. Few studies have examined NTP for generating activation effects on fungal cellular processes without causing mutations. Further research is required to show whether the activation effects on fungi are due to mutations or some other cause.

## 5. Mechanisms of Fungal Inactivation and Activation by NTP

**Figure 2:**
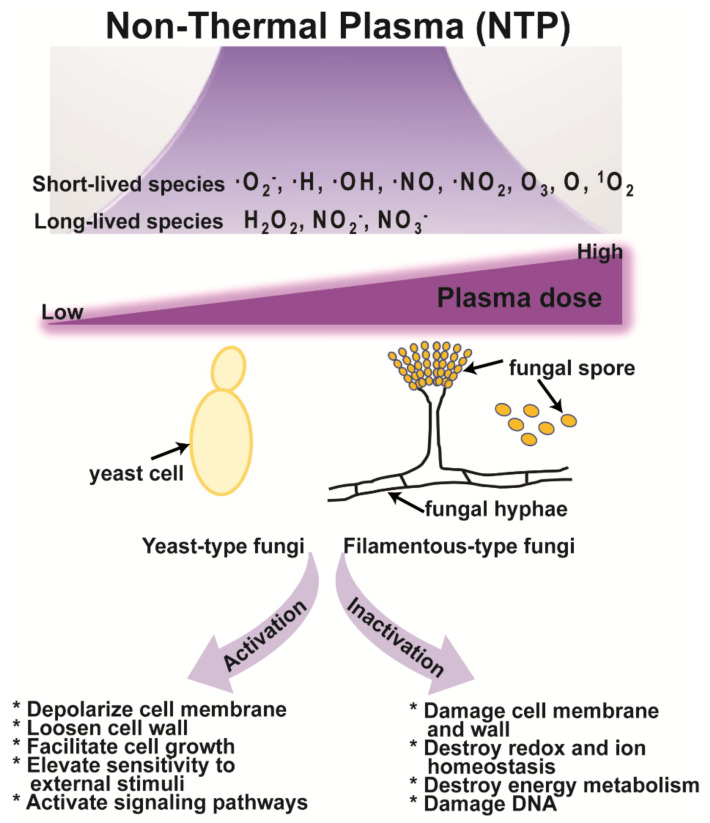
Proposed mechanism of fungal activation and inactivation by NTP.

Many studies have suggested that short- and long-lived reactive species generated by NTP are the main factors that regulate the inactivation and activation of microorganisms, including fungi [18,20,156]. NTP, with an influx of air on liquid surfaces, can generate reactive species, such as free electrons, ·O_2_^−^, ·H, ·OH, ·NO, ·NO_2_, O_3_, atomic oxygen (O), and singlet oxygen (^1^O_2_), which feature relatively short lifetimes [207]. Short-lifetime species produced on liquid surfaces react with species in solution, producing secondary species, such as hydrogen peroxide (H_2_O_2_), nitrite (NO_2_^−^), and nitrate (NO_3_^−^), which can exert a more substantial influence on cells and organisms [208]. Short- and long-lifetime species are responsible for the interaction between plasma and biological objects, and double-edged effects (inactivation and activation) of NTP may result mainly from the action of reactive species (Figure 2). Although the mechanisms underlying the action of NTP are more often reported for bacteria than for fungi, it is assumed that many of the mechanisms may be common [156].

NTP may destroy cell membranes, intracellular redox, ion homeostasis (intracellular H^+^ and K^+^), and energy metabolism (mitochondrial membrane potential, intracellular Ca^2+^, and ATP levels), and damage the DNA (Figure 2). Plasma-generated reactive species interact with proteins to form superoxides or interact with DNA to cause DNA alkylation or inter-chain cross-linking, as well as changing the cell’s metabolic activity and genetic characteristics [209,210]. In yeast, inactivation by NTP produces ·OH and ^1^O_2_. The ·OH radical attacks cell membranes and increases the permeability, while the ^1^O_2_ radical interferes with cell metabolism [211]. Other studies have found that free radicals damaged the cell membranes and walls and could enter cells where they inhibited the normal physiological activities of DNA, RNA, and proteins, eventually killing the microorganisms [212].

The mechanisms of fungal activation by NTP have been rarely studied. NTP can trigger the depolarization of the cell membrane, elevation of calcium influx, and enhancement of secretory vesicle accumulation near the hyphal tips resulting in increased enzyme secretion in a fungus (Figure 2) [20]. In bacteria, charged reactive species generated by NTP penetrate the cell wall under the action of an electric field and make the cell wall looser. This facilitates bacterial cell growth, increases its sensitivity to external stimuli, and activates signaling pathways in bacterial cells [213]. However, the strong and continuous action of charged species on a bacterium can result in the degeneration of the cell membrane, the outflow of the cell lysate, and the death of the bacterium [213]. Similar processes can occur in fungi if the intensity of the plasma is not continuously strong.

## 6. Conclusions and Future Perspectives

Fungi exert a significant impact on human life as agents threatening human health and the ecosystem or by providing benefits to industry. The efficient control and use of fungal resources are advantageous for the economy and industry. Studies performed over the past decade demonstrate that NTP offers great potential as a universal tool for inactivating harmful fungi or activating the functions of beneficial fungi. An enormous amount of data support that NTP can be an efficient and eco-friendly remover of fungi without marked damage on the quality of contaminated and infected objects, thereby replacing chemical fungicides. However, further research is still needed to fine-tune the conditions of NTP to support optimal fungal control on foods and agricultural products and restrain human and animal fungal pathogens. Safety issues related to NTP treatment, such as the generation of genetically and phenotypically modified unfavorable fungal strains, as well as the protection of nearby cells and tissues from plasma treatment, also require further detailed investigations.

A limited number of studies are available on the application of NTP to activating the functional aspects of beneficial fungi. The majority of these studies are focused on using NTP to generate functionally improved mutant strains. Enhancing the functional aspects of fungi without mutations may make NTP a safe and reliable technology. Therefore, future research should focus on addressing this aspect of NTP. In addition, the application of NTP technology to improving the functions of beneficial fungi could create a potential emerging, low-competition market in the food and agriculture industries.

For the productive application of NTP technology, the establishment of a database of fungal responses to various plasma intensities may be essential because a broad spectrum of effects can be obtained based on the different doses or intensities of NTP used.

## Figures and Tables

**Table 1:** Studies showing the use of NTP for fungal inactivation.

Application	Target Fungi (Materials)	Plasma Source (Treatment Parameters)	Effects	Ref.
	Suspension of fungal spores/cells and fungal biofilm			
Inactivation and inhibition of growth	*Alternaria* sp. *Aspergillus oryzae* *Byssochlamys nivea* *Cladosporium sphaerospermum*	Corona discharge plasma (9 kV, 300 µA, filtered air) Dielectric barrier discharge plasma (high-voltage, air)	*C.sphaerospermum*, *A.* sp. and *B. nivea*, *A. oryzae* in order of sensitivity to plasma Spore inactivation time: within 10–40 min	[27]
	*Ascochyta pinodella* *Fusarium culmorum*	Dielectric barrier discharge plasma (20 kV, ~560 W, air)	Complete inhibition of hyphal growth of both fungi after 360 s exposure	[13]
	*Aspergillus brasiliensis*	Plasma activated water (PAW): treated with plasma jet (1.24 kV, 2.96 A, 3.9 W, air)	15% reduction in spore viability after 30 min in PAW	[28]
	*Aspergillus flavus*	RF plasma jet (80–800 kHz RF power, 100 W, mixture of argon Ar and oxygen O_2_)	100% inhibition of growth 48 h after 10 min treatment at 20 W	[29]
		Gaseous plasma and plasma-activated aqueous broth (PAB) Plasma source: surface barrier discharge plasma (5–15 kV at 40 kHz, 0.79, 1.24, 1.62 W/cm^2^, air)	Gaseous plasma treatment: over four log reduction in spore viability after 240 and 480 s treatments under three power conditions PAB treatment: no significant reduction in spore viability	[30]
	*Aspergillus niger* *Penicillium citrinum*	Dielectric barrier discharge plasma (3 kV at 230 Hz on dielectric ceramic electrode, −4 kV on needle electrode, helium He)	Maximum 98–99.9% deactivation of fungal spores after 5 h treatment	[31]
		Microwave plasma jet (2.45 GHz, 1 kW, Ar)	100% inactivation of fungal spores after 1 s treatment	[32]
	*Aspergillus niger* *Cladosporium cladosporioides* *Penicillium citrinum* *Chaetomium* sp.	Microwave plasma jet (2.45 GHz, 1 kW, Ar)	100% inactivation of fungal spores after 1 s treatment	[33]
	*Aspergillus ochraceus* *Penicillium expansum*	Plasma jet (2.5 kV at 25 kHz, 3 mA, 4 W, Ar)	*A. ochraceus*: maximum 3.42 log_10_ CFU reduction after 5 min treatment *P. expansum*: maximum 3.11 log_10_ CFU reduction after 5 min treatment	[34]
	*Aspergillus oryzae* *Cladosporium sphaerospermum* *Penicilium crustosum*	Corona discharge plasma (9.7 kV, 400 µA, filtered air)	99.9–100% spore inactivation after 30 min treatment Significant growth inhibition	[35]
	*Aureobasidium pullulans*	Dielectric barrier discharge plasma (9.3 kV at 11 kHz, Ar)	~100% and ~30% inactivation of non-melanized and melanized cells after 60 min treatment Improvement of fungicidal efficacy of plasma by using FeCl_2_ and FeSO_4_ together	[36]
	*Candida albicans*	Linear microdischarge plasma jet (13.56 MHz, 17 W, He)	Changes in genome sequence, enzyme activity at sublethal dose No change in carbon assimilation and drug susceptibility at sublethal dose	[37]
		Plasma jet (1.8 W, He)	20–30 mm^2^ inhibition zone after 3 min treatment Maximum 11 mm in diameter of inhibition zone after 3 min treatment on five fungal strains	[38,39]
		Plasma activated water (PAW): treated with nanosecond pulsed DBD plasma (50 mJ per pulse, 20 kV at 1000 Hz, air)	100% cells killed after 10–30 min incubation in 5 or 10 min-treated PAW	[40]
		RF plasma jet (15 MHz RF power, 10 kV, mixture of 98% He and 2% O_2_)	31–82% inhibition of growth, 40–91% reduction in ergosterol biosynthesis, 43–57% reduction in biofilm formation and activities of phospholipase and proteinase	[41]
		High-voltage nanosecond pulse plasma jet (6 kV at 1.5 kHz, mixture of 99% He and 1% O_2_	>99.99% inactivation of fungal cells after 30–180 s treatment	[42]
		Dielectric barrier discharge plasma (10 kV at 7.1 kHz, mixture of 99% He and 1% O_2_)	100% cells killed after 5 min treatment	[43]
		Plasma jet (8 kV at 8 kHz, mixture of 97% He and 3% O_2_)	>99.9% inactivation after 3.5 min treatment in the condition of covering A small fraction of fungal cells inactivated even after 8 min treatment without covering	[14]
		Dielectric barrier discharge plasma (30 kV at 60 kHz, air)	100% inactivation of cells after 30 s treatment	[44]
	*Candida parapsilosis* *Magnusiomyces magnusii* *Saccharomyces cerevisiae* *Schizosaccharomyces pombe*	Plasma jet (13 kV at 5 kHz, Ar)	Less than 10% survival of cells after 10 min treatment and 2 h incubation Maximum 0.76% survival of *S. pombe* cells	[45]
	*Cladosporium fulvum*	Plasma jet (5–12 kV at 5–13 kHz, mixture of 99% Ar and 1% O_2_)	Complete killing of fungal spores after 60 s treatment Disruption of membrane and leaking of cytoplasm DNA and protein damage	[46]
	*Colletotrichum gloeosporioides*	Plasma-activated water (PAW): treated with corona discharge plasma jet (3–4 kV at 20 kHz, mixture of 21% O_2_ and 79% N_2_ or 99.99% O_2_)	96% and 56% spore inactivation after 30 min and 10 min incubation, respectively, in PAW generated using air plasma 55% and 15% spore inactivation after 30 min and 10 min incubation, respectively, in PAW generated using oxygen plasma	[47]
	*Cordyceps bassiana*	Plasma jet (22 kHz, 9 W, Ar)	16.4% spore survival after 5 min treatment Reduction in DNA content and alteration to cell wall	[48,49]
		Electric shock-free plasma jet (67 kHz, air)	0.8% spore survival after 6 min treatment Plasma generated H_2_O_2_ and NOx as major players in antifungal activity	[50]
	*Fusarium graminearum* *Fusarium oxysporum* *Neurospora crassa*	Microwave plasma jet (2.45 GHz, 1.6 W, mixtures of 83% Ar and 17% O_2_, 83% Ar and 17% N_2_, or 83% Ar and 17% air, 100% N_2_)	Dramatic reduction in fungal hyphal growth when O_2_ is used in plasma generation Increased growth inhibition with increased power and pulse length	[51]
	*Fusarium oxysporum* f. sp. *lycopersici*	Dielectric barrier discharge plasma (discharge at 0.75 kV and 80 mA, 7.5 W, air or Ar)	<10% survival of fungal spores after treated in saline for 10 min and then incubated for 6 h Increase in size of lipid droplets inside spore cell and number of apoptotic spores	[52]
	*Neurospora crassa*	Plasma jet (4 kV at 22 kHz, 13 mA, Ar)	~80% reduction in spore viability after 3 min treatment in water Less effective when treated in saline and culture media Crushed spores, cell wall damage, degradation of β-carotene	[53,54,55]
	*Penicillium digitatum*	Plasma jet (discharge at −500–−1000 V and 3–16 mA, 5 W, humid air)	Maximum 91% spore inactivation after 9 min treatment and moisture was added in working gas	[56]
		Plasma generated oxygen radical source (Tough Plasma; Fuji Machine MFG Co. Ltd., Chiryu, Japan)	>2 log_10_ CFU reduction in spore viability after 5 min treatment at distance of 10 mm	[57,58]
		Microwave plasma jet (2.45 GHz, 50 W, O_2_)	~2 log_10_ CFU reduction in spore viability after 10 min treatment Decay of electron spin resonance (ESR) signal in situ from plasma treated spores	[59]
		Plasma jet (6 kV at 60 Hz, Ar)	>3 log_10_ CFU reduction in spore viability after 7 min treatment at distance of 10 mm	[60]
	*Penicillium* sp.	Plasma microdischarge torch (discharge at 5–10 kV and 15 mA, 7.5–15 W, air)	Inhibition of fungal growth after treatment at distance of 3 cm Partial spore damage after plasma treatment	[61]
	*Saccharomyces cerevisiae*	Dielectric barrier discharge plasma (Ar)	~99% reduction in cell viability after 10–15 min treatment Accumulation of oxidative stress responsive transcription factor, mitochondrial fragmentation, enhanced intracellular oxidation	[62]
		Plasma jet (4 kV at 22 kHz, 13 mA, Ar)	~100% reduction in cell viability after 2 min treatment in water Less effective when treated in saline and culture media Crushed cells, increased lipid peroxidation, and DNA degradation after plasma treatment in water and saline	[63]
		Dielectric barrier discharge plasma (12 kV at 20 kHz, 3.8 mA, 26 W, air)	Maximum ~2 log_10_ CFU reduction in cell viability after 5 min treatment Release of protein and nucleic acids, cell cycle arrest at G1 phase	[64]
		Plasma microjet (0.56 kV, 30 mA, mixture of 98% He and 2% O_2_)	Maximum 100% reduction in cell viability after 5 min treatment	[65]
		Plasma microjet (0.56 kV, 30 mA, mixture of 98% He and 2% O_2_)	>2 log_10_ CFU reduction in cell viability after 5 min treatment in water ROS and acidic pH exert a synergistic antimicrobial effect	[66]
		Surface micro-discharge plasma (8 kV at 8 kHz, 0.07 W/cm^2^, He)	Inactivation pattern of cells is dependent on distribution and concentration of OH radical	[67]
		Plasma microjet (0.56 kV, 30 mA, mixture of 98% He and 2% O_2_)	Plasma-generated ROS leads to the accumulation of intracellular ROS and Ca^2+^, which in turn cause apoptosis of yeast cells	[68]
		Plasma microjet (discharge at 0.56 kV and 30 mA, mixture of 98% He and 2% O_2_)	Evaluated the protection effects of gene manipulation and reactive species scavengers against plasma-induced oxidative stresses: overexpression of superoxide dismutases reduces plasma oxidative stress	[69]
	*Trichophyton rubrum*	Plasma jet (10 kV at 15 MHz, 10 W, mixture of 98% He and 2% O_2_)	~91% spore inactivation, >50% reduction in fungal dry weight, and 53% inhibition in ergosterol synthesis after 3 min treatment	[70]
Inhibition of biofilm formation	*Aspergillus flavus*	Gaseous plasma and plasma activated water (PAW) Plasma source: dielectric barrier discharge plasma (80 kV, air)	Maximum 2.2 and 0.6 log_10_ CFU reduction in spore viability after treatment with gaseous plasma and plasma activated water, respectively ~50% reduction in biofilm biomass after gaseous plasma treatment	[71]
	*Candida albicans*	Plasma jet (15 kV at 1 kHz, mixture of 99.5% He and 0.5% O_2_)	Reduction from 35.6 × 10^2^ CFU/mL to 4.6 × 10^2^ CFU/mL after 8 min treatment of suspension Complete killing of fungal cells in biofilm after 8 min treatment	[72]
		Plasma jet (1.8 W, He)	40 times reduction in filamentation Reduction in fungal adherence and biofilm viability (~1 log_10_ CFU reduction in cell viability within biofilm) No effect on exoenzyme production	[73]
		Surface dielectric barrier microdischarge plasma (9 kV at 1 kHz, 0.02 W/cm^2^, air)	3–5 log_10_ CFU reduction in cell viability within biofilm	[74]
		Plasma jet (kINPen08; 2–6 kV at 1.7 MHz, 65 W, Ar, mixture of 99% Ar and 1% O_2_)	Removal of biofilm with a thickness of 10 to 20 µm within 300 s plasma treatment using mixture of Ar and O_2_ as working gas Insufficient removal of biofilm using Ar plasma	[75]
		Plasma microjet (discharge at 0.56 kV and 30 mA, mixture of 98% He and 2% O_2_)	Complete removal of biofilms after 1 min treatment Severe deformation of fungal elements	[76]
		Plasma jet (kINPen09; 2–6 kV at 1.82 MHz, Ar, mixture of Ar and O_2_) Hollow electrode dielectric barrier discharge (HDBD) plasma (37.6 kHz RF power, 9 W, 9 kV, Ar, mixture of Ar and O_2_) Volume dielectric barrier discharge (VDBD) plasma (10 kV at 40 kHz, 16 W, Ar)	kINPen09; maximum 1 log_10_ CFU reduction in cell viability within biofilm HDBD; maximum 3.3 log_10_ CFU reduction in cell viability within biofilm VDBD; maximum 5.2 log_10_ CFU reduction in cell viability within biofilm	[77]
	**Fungal contamination in agriculture and foods**			
Disinfection of seeds	*Alternaria alternata* *Aspergillus flavus* *Fusarium culmorum* (maize seeds)	Diffuse coplanar surface barrier discharge plasma (80 W/cm^3^, air)	Reduction of 3.79 log_10_ CFU/g in *F. culmorum* after 60 s treatment Reduction of 4.21 log_10_ CFU/g in *A. flavus* and 3.22 log_10_ CFU/g in *A. alternata* after 300 s treatment Increase in seed surface wettability Enhancement of seedling growth	[78]
	*Alternaria alternata* *Alternaria botrytis* *Aspergillus brasiliensis* *Epicoccum nigrum* *Fusarium culmorum* *Fusarium poae* *Gibberella zeae* *Mucor hiemalis* *Penicillium* sp. *Rhizopus stolonifer* *Trichoderma* sp. (winter wheat)	Plane-type plasma (8 kV at 0.1–83 kHz, air)	Reduction in number of fungal colonies on seeds after 10 s treatment Positive effect on seed germination and initial seedling development	[79]
	*Aspergillus clavatus* *Aspergillus flavus* *Fusarium culmorum* *Fusarium nivale* *Trichothecium roseum* (wheat)	Diffuse coplanar surface barrier discharge plasma (100 W/cm^3^, air)	Order of efficiency in fungal decrease after plasma treatment; *F. nivale* > *F. culmorum* > *T. roseum* > *A. flavus* > *A. clavatus*	[80]
	*Aspergillus flavus* *Aspergillus parasiticus* (groundnuts)	RF plane-type plasma (13.56 MHz RF power, 40 W and 60 W, air)	97.9% and 99.3% reduction in CFU of *A. parasiticus* and *A. flavus*, respectively, when treated at 60 W	[81]
	*Aspergillus niger* *Penicillium decumbens* (lentil seeds)	Diffuse coplanar surface barrier discharge plasma (RPS400; 400 W, air)	1.6 and 3.1 log_10_ CFU/g reduction for *A. niger* and *P. decumbens*, respectively, after 10 min treatment No significant effect on germination	[82]
	*Aspergillus parasiticus* (hazelnuts, peanuts, pistachio nuts)	Low-pressure plasma (20 kV at 1 kHz, 300 W, 100 or 500 mTorr, air or SF_6_)	1 and 5 log_10_ CFU reduction after 5 min treatment with air and SF_6_ plasma, respectively	[83]
	*Aspergillus* sp. *Penicillium* sp. (black beans)	Dielectric barrier discharge plasma (8 kV, 510 W, air)	Complete fungal disinfection after treatment for at least 10 min Wrinkling on seed surface and change in cotyledon color	[84]
	(seeds of tomato, wheat, bean, chickpea, soybean, barley, oat, rye, lentil, corn)	Low-pressure plasma (20 kV at 1 kHz, 300 W, air or SF_6_ at low pressure)	Fungal decontamination below 1% of initial load 3 log_10_ CFU reduction after 15 min treatment with SF_6_ plasma No significant change in seed germination quality	[85]
	*Cladosporium cucumerinum* *Didymella bryoniae* *Didymella licopersici* (cucumber and pepper seeds)	Surface dielectric barrier discharge plasma (20 kV at 15 kHz, 400 W, air)	No presence of *C. cucumerinum* and 60–80% reduction in *D. bryoniae* spore viability on cucumber seeds after 20 s treatment 50–80% reduction in *D. lycopersici* spore viability on pepper seeds after 4 s treatment Improvement in seed germination	[86]
	*Cladosporiumfulvum* (tomato seeds)	Plasma jet (5–12 kV at 5–13 kHz, mixture of 99% Ar and 1% O_2_)	Maximum 14% reduction in seed rotting caused by fungal infection after 60 s treatment	[46]
	*Clonostachys rossmaniae* *Coniochaeta fasciculat* *Cylindrocarpon destructans* *Fusarium proliferatum* *Humicola fuscoatra* *Mortierella hyalina* *Pyrenochaeta* sp. (ginseng seeds)	Dielectric barrier discharge plasma (120 V at 60 Hz, Ar or mixture of 80% Ar and 20% O_2_)	27.7% and 40% survival of fungal spores on seeds after Ar and Ar/O_2_ plasma treatments, respectively	[87]
	Contaminated fungi (Pak-Choi seeds)	Corona discharge plasma jet (20 kV at 58 kHz, 1.5 A, air)	1.3–2.1 log_10_ CFU/g reduction after 3 min treatment Positive effect on seed germination after treatment for up to 2 min	[88]
	(sweet basil seeds)	Surface dielectric barrier discharge plasma (8.6 kV at 500 Hz, 6.5 W, air)	~30% reduction in number of seeds naturally contaminated with fungi after 300 s treatment Improvement in growth of seedlings	[89]
	(broccoli seeds)	Corona discharge plasma jet (20 kV at 58 kHz, 1.5 A, air)	1.5 log_10_ CFU/g reduction of natural fungal flora on seeds after 3 min treatment Positive effect on seed germination and seedling growth after treatment for up to 2 min	[90]
	(rice seeds)	Microcorona dielectric barrier discharge plasma (~14 kV at ~700 Hz, air)	Complete removal of fungal contamination from seeds after 1 min treatment and then incubation for 14 days Enhancement of seed germination	[91]
	(barley and corn seeds)	Glow discharge low pressure plasma (100 or 200 W, 15 Pa, air)	Barley: 25% reduction in fungal contamination on seeds after 20 min treatment, retardation of seed germination and no influence on seedling growth Corn: ~40% reduction in fungal contamination on seeds after 10 min treatment, no influence on seed germination and slight improvement of seedling growth	[92]
	*Diaporthe/Phomopsis* (D/P) *complex* (soybean seeds)	Dielectric barrier discharge plasma (65 or 85 W, 50 Hz, ~50 mA, N_2_ or O_2_)	~49–81% disinfection of seeds Significant stimulating effects on seed germination and vigor	[93]
	*Fusariumcircinatum* (pine seeds)	Diffuse coplanar surface barrier discharge plasma (10 kV at 14 kHz, 400 W, air)	14–100% disinfection of seeds after treatment up to 300 s Reduction in seed germination percentage	[94]
	*Fusarium fujikuroi* (rice seeds)	Plasma jet (20 kV at 10 kHz, humid air)	Reduction to 7.8% of non-treated control in the percentage of plants with disease symptoms after 10 min treatment of seeds in water No adverse effect on seed germination and seedling growth	[95]
		Underwater arc discharge plasma (10 kV at 12 Hz, air in water)	~80% disinfection of seeds after 20 min treatment	[96]
		Dielectric barrier discharge plasma (30 kV at 22 kHz, air)	>92% disinfection of seeds after 120 s treatment Significant reduction in disease development after 10 min treatment on seeds No adverse effect on seed germination and seedling growth	[97]
	*Fusarium oxysporum* (Scots pine seeds)	Diffuse coplanar surface barrier discharge plasma (20 kV at 14 kHz, 400 W, air)	~6% disinfection of seeds after 3 s treatment Slight increase in seed germination percentage	[98]
	*Penicillium verrucosum* (wheat and barley seeds)	Dielectric barrier discharge plasma (80 kV at 50 Hz, air)	Maximum 2.1 and 2.5 log_10_ CFU/g reduction in barley and wheat seeds, respectively, after 20 min treatment followed by incubation for 24 h No significant effect on seed germination	[99]
	*Rhizoctonia solani* (brassicaceous seeds)	Atmospheric pressure plasma (10 kV at 10 kHz, Ar) Low pressure plasma (5.5 kV at 10 kHz, 80 torr, Ar)	Atmospheric-pressure plasma: 97% reduction in fungal survival on seeds after 10 min treatment, delay of seed germination Low-pressure plasma: 81% reduction in fungal survival on seeds after 10 min treatment, no change in seed germination rate	[100]
Disinfection of post-harvest vegetables and fruits	*Aspergillus flavus* *Aspergillus parasiticus* (hazelnut)	Atmospheric pressure plasma jet (25 kHz, 655 W, air or N_2_) Low pressure RF plasma (13.56 MHz RF power, 100 W, 0.25 mbarr, air, N_2_ or O_2_)	Atmospheric-pressure plasma: 5.5 and 5.4 log_10_ CFU/g reduction in *A. parasiticus* and *A. flavus* on hazelnuts, respectively, after 1.7 min treatment Low-pressure plasma: 5.6 and 4.7 log_10_ CFU/g reduction in *A. parasiticus* and *A. flavus* on hazelnuts, respectively, after 30 min treatment	[101]
	(hazelnut, maize)	Fluidized bed plasma (5–10 kV at 18–25 kHz, 655 W, air or N_2_)	Maximum 4.09–4.19 and 4.17–4.50 log_10_ CFU/g reduction in *A. parasiticus* and *A. flavus* on hazelnuts, respectively, after 5 min treatment with air plasma, no or little fungal regrowth during storage for 30 days Maximum 5.20 and 5.48 log_10_ CFU/g reduction in *A. parasiticus* and *A. flavus* on maize, respectively, after 5 min treatment with air plasma, no fungal regrowth during storage for 30 days	[102,103,104]
	*Aspergillus niger* (black pepper, allspice berry, juniper berry)	Microwave plasma (2.45 GHz, 600 W, Ar)	Partial inactivation of *A. niger* Reduction in water activity Enhancement of extractability of phenolics or piperine from black pepper	[105]
	(date palm fruit discs)	Plasma jet (25 kV at ~25 kHz, Ar)	Complete removal of fungal spores on fruit discs after 7.5 min treatment with Ar flow 3.5 L/min	[106]
	*Aspergillus niger* *Penicillium italicum* (fruit washwater)	Plasma jet (25 kV at ~25 kHz, Ar)	74.7–100% removal of fungal contamination in the washwater of cherries after 7.5 min treatment	[107]
	*Aspergillus oryzae* *Penicillium digitatum* (rice, lemon)	Surface dielectric barrier discharge (7–10 kV at 10 kHz, air)	~90% and ~100% removal of fungal contamination on rice and lemon surface, respectively, after 20 min treatment	[108]
	*Botrytis cinerea* (blueberry)	Dielectric barrier surface discharge plasma (4 kV at 8 kHz, 5 W, air)	Inhibition of native microbial growth and natural decay of blueberries after plasma treatment Maximum ~40% reduction in decay incidence in blueberries inoculated with *B. cinerea* after 20 min treatment and 10-day storage Minor effects on blueberry quality after less than 15 min treatment but severe oxidative damage to the blueberry peels after 20 min treatment	[109]
	*Botrytis cinerea* *Monilinia fructicola* (cherry)	Surface dielectric barrier discharge plasma (8.6 kV at 500 Hz, 6.5 W, air)	>50% reduction in number of infected fruits after 5 min treatment in earlier days Pre-treatment of fruits by plasma before inoculation enhances the resistance to infections	[15]
	*Colletotrichum gloeosporioides* (mango)	Gliding arc discharge (discharge at 8 kV and 0.6 A, 600 W, humid Ar)	Significant inhibition of mycelium growth Significant delay in disease development in mango after 7 min treatment during storage for 12 days at 30 °C	[110]
	Contaminated fungi (blueberry)	Dielectric barrier discharge plasma (discharge at 36 V and 1.8 A, air)	25.8% decrease in fungal contamination on blueberry and 5.2% blueberry decay rate after 10 min treatment during storage for 20 days	[111]
	(mung bean sprout)	Plasma-activated water (PAW): treated with plasma jet (5 kV at 40 kHz, 750 W, air)	2.84 log_10_ CFU/g reduction in yeasts and molds on mung bean after 30 min treatment in PAW No significant change in total phenolic and flavonoid contents and sensory characteristics of mung bean	[112]
	(kumquat)	Corona discharge plasma jet (8 kV at 20 kHz, air)	0.77–1.57 log_10_ CFU/g reduction in yeasts and molds on kumquat after 2 min treatment No significant change in taste, flavor, color, texture, and total acceptance	[113]
	(button mushroom)	Plasma activated water (PAW): treated with plasma jet (18 kV at 10 kHz, mixture of 98% Ar and 2% O_2_)	0.5 log_10_ CFU reduction in fungi on mushroom during storage for over 7 days Delay in mushroom softening No significant change in color, pH, antioxidant properties	[114]
	(blueberry)	Plasma jet (47 kHz, 549 W, air)	1.5–2.0 log_10_ CFU/g reduction in yeasts and molds on blueberries after 7 days Significant reduction in firmness and anthocyanin content after treatment for over 60 s	[115]
	(banana, grape)	High-field plasma system (2 kV at 500 Hz, 20–30 µA, 3–4 × 10^6^ V/m electric field, air)	No increase in mold load on surface of fruits during storage in high-filed plasma system Lower amount of ethylene gas emitted during storage in high-field plasma system	[116]
	*Fusarium oxysporum* (paprika)	Plasma jet (28 kHz, 1000 W, air)	50% inhibition of fungal growth on paprika after 90 s treatment No significant change in color and hardness during 14 days of storage	[117]
	*Penicillium digitatum* (citrus)	Dielectric barrier discharge plasma (10 kV at ~10 kHz, air)	~90% and ~99% reduction in CFU number of fungal spores on citrus surface after 1 s and 3 s treatments, respectively	[118]
	*Penicillium italium* (mandarin)	Microwave plasma jet (2.45 GHz, 900 W, 500–30,000 Pa, N_2_)	84% reduction in disease incidence after 10 min treatment Significant increase in total phenolic content and antioxidant activity of mandarin peel	[119]
	*Penicillium venetum* (citrus)	Roller conveyor type dielectric barrier discharge plasma (11.87 kV at 8.85 kHz, air)	~0.7–1 log_10_ CFU/mL reduction in viable spore number after 2 min treatment	[120]
Disinfection of pre-harvest plants	*Botrytis cinerea* (cannabis influorescence)	RF plasma (6 kV, low pressure air with the addition of H_2_O_2_ (35%))	5 log_10_ CFU reduction in viable fungal spores on influorescence	[121]
	*Colletotrichum gloeosporioides* (Green Emerald leaves)	Plasma jet (5 kV, 11 W, mixture of 97% He and 3% O_2_)	Complete recovery of leaves with black spot diameter of <2 mm after plasma treatment for 3 weeks (twice a day and 10 s per each treatment)	[122]
Food sanitation	*Aspergillus brasiliensis* (oninon powder)	Microwave plasma (2.45 GHz, 900 W, He)	1.6 log_10_ CFU/cm^2^ reduction after 40 min treatment at 400 W	[123]
	*Aspergillus flavus* (in-package beef jerky)	Flexible thin-layer plasma (15 kHz, air)	2 -3 log_10_ CFU/g reduction in number of viable fungal spores on beef jerky after 10 min treatment No significant change in metmyoglobin content, shear force, myofibrillar gragmentation index Negative effects on flavor, off-color, and overall acceptability	[124]
	(in-package pistachio)	Dielectric barrier discharge plasma (12.5 kHz suppressed by a modulated pulsed signal at 110 Hz, 2.49 W/cm^3^, air)	4 log_10_ CFU/sample reduction in number of viable fungal spores on pistachio after 18 min treatment Slight reduction in moisture content of pistachio and no change in pH of pistachio	[125]
	(red pepper powder)	Microwave plasma (2.45 GHz, 50–1000 W, N_2_, mixture of N_2_ and O_2_, He, or mixture of He and O_2_)	2.5 log_10_ CFU/g reduction in number of viable fungal spores in red pepper powder after 20 min treatment with N_2_ plasma	[126]
	(brown rice cereal bar)	RF plasma jet (50–600 kHz RF power, 0–40 W, Ar)	No fungal growth on cereal bars for up to 20 days under 25 °C and 100% relative humidity after 20 min treatment at 40 W	[127]
	*Aspergillus* sp. *Rhizopus* sp. *Penicillium* sp. (saffron)	Low-pressure RF oxygen plasma (10–90 W, 8.5 mTorr system pressure, 13.5 mTorr working pressure, O_2_)	Complete inactivation of fungi after 15 min treatment at 60 W	[128]
	*Candida albicans* *Saccharomyces cerevisiae* (tomato juice)	AC gliding arc plasma (3.8 kV at 50 Hz, 40 W, N_2_)	~4 log_10_ CFU/g reduction in fungal cell viability in tomato juice after 600 s treatment followed by storage for 10 days No substantial change in the physicochemical properties of tomato juice	[129]
	*Cladosporium cladosporioides* *Penicillium citrinum* (dried filefish fillets)	Oxygen plasma (photoplasma; Model InDuct, ID 60, BioZone Scientific International Inc., Orlando, FL, USA)	0.91 and 1.04 log_10_ CFU/g reduction in number of *C. cladosporioides* and *P. citrinum* on fillets, respectively, after 3–20 min treatment Increase in the level of thiobarbituric acid reactive substance (TBARS) and decrease in overall sensory acceptance after 20 min treatment	[130]
	Contaminated fungi (shredded salted kimchi cabbage)	Plasma-activated water (PAW): treated with a plasma system (18 kV at 14.3 kHz, air)	1.8 log_10_ CFU/g reduction in yeasts and molds associated with kimchi cabbages after submerging in PAW treated with plasma for 120 min Combined treatment with mild heating can enhance fungal inactivation	[131]
Mycotoxin degradation	Aflatoxin (hazelnuts)	Dielectric barrier discharge plasma (100–150 kHz, 0.4–2 kW, N_2_ or mixture of N_2_ and air)	>70% reduction in the content of total aflatoxins and aflatoxin B1 on hazelnuts after 12 min treatment at 1000 W	[132]
	(groundnuts)	RF-plane-type plasma (13.56 MHz RF power, 40 W and 60 W, air)	>70% and 90% reduction in the content of aflatoxin B1 on groundnuts after treatment for 50 min at 40 W and 12 min at 60 W, respectively	[81]
	(hazelnuts)	Atmospheric pressure plasma jet (25 kHz, 655 W, air)Low-pressure RF plasma (13.56 MHz RF power, 100 W, <0.25 mbar, air)	72–73% reduction in the amount of aflatoxin B1 spiked on hazelnuts after treatment with both plasmas	[133]
	(rice and wheat)	Corona discharge plasma jet (20 kV at 58 kHz, air)	45–56% reduction in the level of aflatoxin B1 on rice and wheat after 30 min treatment	[134]
	(corn kernels)	DC surface barrier discharge plasma (0.18–0.31 W/cm, air)	Complete degradation of aflatoxin B1 after 480 s treatment	[135]
	(slideglass, pistachio nuts)	Dielectric barrier discharge plasma (15 kV at 20 kHz, 130 W, air)	Maximum 64.63% and 52.42% reduction in the level of aflatoxin B1 on slideglass and pistachio nuts, respectively, after 180 s treatment	[136]
	Deoxynivalenol, zearalenone, enniatins, fumonisin B1, T2 toxin, sterigmatocystin, AAL toxin (coverglass, rice extracts)	High-voltage pulsed atmospheric-pressure-plasma (~19 kV at 17 kHz, air)	Complete removal of all mycotoxins on coverglass after 60 s treatment; fumon: fumonisin B1 is most sensitive and sterigmatocystin is most resistant Various degradation rates of mycotoxins in extracts of fungal cultures on rice	[137]
	**Fungal contamination in medicine**			
Prevention of onychomycosis	*Candida albicans* *Trichophyton mentagrophytes* (fungal suspension, infected nail)	Dielectric barrier discharge (5–20 kV at 1 Hz–1 kHz, air)	Complete killing of *C. albicans* and T. *mentagrophytes* in suspension after 12 min treatment at dose of 30 and 15 kPulses, respectively 100× reduction in viable cell number of *C. albicans* on nail after treatment at dose of 550 kPulses	[138]
	*Trichophyton benhamiae* *Trichophyton interdigital* *Trichophyton rubrum* (fungal suspension, patients with infected nails)	Negative DC corona discharge (7 kV, 150 µA, air)	Complete inactivation of all fungal species in vitro straight after plasma treatment More than 70% of onychomycosis patients are cured after the combined treatment of plasma and nail plate abrasion and refreshment	[139]
	*Trichophyton rubrum* (infected sliced hoof discs)	Plasma jet (8 kV at 4 kHz, mixture of 99.5% He and 0.5% O_2_) Surface microdischarge (SMD) plasma (2.5 kV at ~25 kHz, air) Floating electrode (FE) dielectric barrier discharge (DBD) plasma (6 kV at 4 kHz, air)	1 and ~3 log_10_ CFU reduction in viable cell number of *T. rubrum* infected in hoof discs after 45 min treatment with SMD plasma and 10 min treatment with FE-DBD plasma, respectively	[140]
Prevention of dermatophytosis	*Arthroderma benhamiae* *Microsporum gypseu* *Trichophyton interdigitale* *Trichophyton rubrum* (fungal suspension in water, fungal spores on agar plates)	Positive and negative point-to-plane corona discharge plasma (10 kV, 0.5 mA, air) Cometary discharge plasma (10 kV at 20 kHz, air)	In suspension: significant decrease in number of viable spores of all fungal species after 15 min treatment, complete killing of *T. interdigitale* and *T. rubrum* spores after 25 min treatment On agar plates: complete killing of all fungal species, except *M. gypseu*, after 25 min treatment	[141]
	*Candida albicans* *Candida glabrata* *Candida krusei* (fungal suspension in water, fungal spores on sabouraud dextrose agar plates)	Plasma microjet (400 V, 35 mA, mixture of 98% He and 2% O_2_)	>90% inactivation of fungal spores after 10 min on agar plates and 1 min in suspension	[142]
	*Epidermophyton floccosum* *Microsporum canis* *Microsporum gypseum* *Trichophyton mentagrophytes*, *Trichophyton rubrum* (fungal suspension, infected guinea pig)	Plasma jet (0.6 kV, 15 mA, 21 kHz, air) in combination with silver nanoparticles	Reduction in values of minimum inhibitory concentration (MIC) of silver nanoparticles after the combined treatment with plasma Enhancement of fungal mycelium permeability of nanoparticles after the combined treatment with plasma Increase in efficiency of healing and suppressing disease symptoms of guinea pig skin after the combined treatment of nanoparticles and plasma	[143]
Prevention of dermatophytosis	*Trichophyton mentagrophytes* (Infected guinea pig)	Cometary discharge plasma (5 kV, discharge at 50–100 µA, air)	A week shorter and milder infection in guinea pigs treated with plasma Significant reduction in number of viable fungal cells in guinea pigs treated with plasma No adverse effects on guinea pigs	[144]
Prevention of oral candidiasis	*Candida albicans* (Fungal biofilm, infected mouse tung)	Amplitude-modulated cold atmospheric-pressure plasma jet (13 kV, 32 kHz, He)	Significant reduction in the viability of *C. albicans* biofilms after 5 min treatment No significant difference in values of CFU/tongue but marked reduction in candidal tissue invasion after plasma treatment No adverse effects on mouse cells	[145]
Killing of clinical fungal strains	*Candida albicans* *Microsporum canis* *Trichophyton interdigitale* *Trichophyton rubrum* (fungal cells on agar plates, dandruffs, shoes from a patient with chronic tinea pedis)	Plasma jet (1–5 kV and 1.5 MHz RF power, Ar)	The largest growth inhibition zone on *C. albicans* agar plate and the smallest zone on *M. canis* agar plate after 15 s treatment Complete removal of viable fungal elements of *T. interdigitale* in dandruffs and contaminated shoes after plasma treatment	[146]
	*Candida albicans* (fungal cells on agar plates)	Glow discharge microplasma jet (1 kV at 20 kHz, 860 Torr, He)	Increase in growth inhibition zone in sabouraud dextrose agar plates after 1.5 min treatment	[147]
	*Trichophyton mentagrophytes* *Trichophyton rubrum* (Fungal suspension, infected skin model)	Floating electrode-dielectric barrier discharge plasma jet (8 kV, ~33 mA, 49 W, Ar)	~96% and 90% reduction in CFU number of *T. mentagrophytes* and *T. rubrum*, respectively, after 5 min treatment Significant inhibition of hyphal growth of both fungal species in infected skin mimicking model after plasma treatment	[148]

**Table 2:** Studies showing the use of NTP for fungal activation.

Application	Fungi	Plasma Source (Treatment Parameters)	Effects	Ref.
Enhancement of spore germination and protein secretion	*Aspergillus oryzae*	Micro-dielectric barrier discharge plasma (1.2 kV, 50–63 mA, 28.8 ms on and 160 ms off pulse times, N_2_)	Significant increase in percentage of spore germination in phosphate buffered saline (PBS) and potato dextrose broth (PDB) after 2 min and 5 min treatments, respectively 7.4–9.3% increase in activity of α-amylase in PDB after 24 and 48 h of plasma treatment (5 min)	[20,168]
		Plasma jet (~0.68 kv at ~83 kHz, ~77 mA, air)	~10% increase in spore germination after 5 min and 10 min treatments Significant elevation of α-amylase activity in PDB after 24–96 h of plasma treatment (10 and 15 min)	[169]
	*Pichia pastoris*	Plasma jet (0–15 kV at 10 kHz, He)	Increased production of recombinant phytase by *P. pastoris* after plasma treatment 125% increase in activity of phytase in commercial enzyme solution after plasma treatment	[18]
Mutagenesis	*Aspergillus nidulans*	Atmospheric and room temperature plasma (ARTP) mutation system: radio-frequency atmospheric-pressure glow discharge (RF APGD) plasma jet (150–300 V, 15–50 MHz RF power, 40–120 W, He) Commercial product from Siqingyuan Biotechnology Co., Ltd., Beijing or Wuxi, China)	Mutant: echinocandin B production of 1.3-fold higher than that of the parental strain	[170]
	*Aspergillus niger*	ARTP mutation system	Four mutants: gluconate production of 15.5%, 32.8%, 12.1%, and 70% higher than that of the parental strain	[171,172]
	*Aspergillus oryzae*	ARTP mutation system	Mutants: 54.7, 17.3, and 8.5% increase in activities of acid protease, neutral protease, and total protease, respectively, 292.3% increase in kojic acid production, enhanced activities of salt-tolerant proteases	[19,173,174]
	*Aspergillus terreus*	ARTP mutation system	Mutant: growth and secretion of itaconic acid in undetoxified enzymatic hydrolysate	[175]
	*Auerobasidium pullulans*	ARTP mutation system	Mutant: 13.8% increase in polymalic acid production	[176]
	*Blakeslea trispora*	ARTP mutation system	Mutant: 55% increase in lycopene production and requirement of 10% less (than that of parent strain) dissolved oxygen for maximum production	[177]
	*Candida glabrata*	ARTP mutation system	Mutant: 32.2–35.4% increase in pyruvate production	[178,179]
	*Candida parapsilosis*	ARTP mutation system	Mutant: 53.98% increase in D-arabitol production	[180]
	*Candida tropicalis*	ARTP mutation system	Mutant: 22% increase in xylitol production, increase in gene expression and activity of xylose reductase	[181]
	*Fusidium coccineum*	ARTP mutation system	Mutant: 59.4% increase in fusidic acid production	[182]
	*Ganoderma lingzhi*	Dielectric barrier discharge plasma (10–15 kV, Ar or He)	Mutant: 25.6% increase in polysaccharides production	[183]
	*Glarea lozoyensis*	ARTP mutation system	Mutant: 1.39 fold increase in pneumocandin B_0_ production	[184]
	*Hericium erinaceus*	ARTP mutation system	Mutant: 22% and 16% increase in the yield of fruiting body and polysaccharide production, respectively	[185]
	*Mortierella alpina*	ARTP mutation system	Mutant: 40.61% increase in arachidonic acid production	[186]
	*Penicillium oxalicum*	Combined treatment with ARTP mutation system and ethylmethanesulfonate	Mutant: 61.1% increase in production of raw starch-degrading enzymes	[187]
	*Pichia anomala*	ARTP mutation system	Mutant: 32.3% increase in sugar alcohol production	[188]
	*Rhodosporidium toruloides*	ARTP mutation system	Mutants: improvement in tolerance to the inhibitory compounds in lignocellulosic hydrolysate and producing lipids with sugarcane bagasse hydrolysate as carbon source, improvement in production of lipids and carotenoids Enhanced expression of four genes is related to the tolerance to lignocellulosic hydrolyzate	[189,190,191,192]
	*Rhodotorula mucilaginosa*	ARTP mutation system	Mutant: 67% increase in carotenoids production	[193]
	*Saccharomyces cerevisiae*	ARTP mutation system	Mutant: 72.54% decrease in production of methanol, which is a toxic by-product of brewing wine Mutant: 56.76% increase in glutathione production, improvement of the activity of glutathione synthetases	[194,195]
	*Sanghuangporous sanghuang*	ARTP mutation system	Mutant: 1.2–1.5 fold increase in polysaccharides production	[196]
	*Starmerella bombicola*	ARTP mutation system	Mutants: over 30% increase in lactonic, acidic, or total sophorolipid production	[197]
	*Trichoderma reesei*	ARTP mutation system	Mutant: increase in cellulase production Mutation in galactokinase gene may be related to improvement of cellulase production Up-regulation of cellulase and hemicellulose genes	[198]
	*Trichoderma viride*	ARTP mutation system	Mutant: 2.18–2.61 fold increase in activities of cellulases Mutant: 1.97 fold increase in total cellulase activity	[199,200]
	*Yarrowia lipolytica*	ARTP mutation system	Mutant: 45.4–51.8% increase in α-ketoglutaric acid production Mutations in genes regulating mitochondrial biogenesis and energy metabolism and a gene associated with cell cycle control are responsible for improvement of α-ketoglutaric acid production Mutant: the highest yield of erythritol production (64.8 g/L erythritol from 100 g/L glycerol)	[201,202,203]

## Data Availability

Not applicable.
